# Melatonin Extends the Edible Window Period of Kiwifruit by Modulating Energy Metabolism and Respiration: A Comparison with 1-Methylcyclopropene and Acetylsalicylic Acid

**DOI:** 10.3390/foods15193489

**Published:** 2026-09-29

**Authors:** Xiaomao Li, Zulin Mei, Nanxin Zhang, Xianzhi Liu, Jiqing Lei, Yuanyuan Liu, Bangdi Liu, Pufan Zheng, Cunkun Chen, Guangjing Chen, Ning Ji, Rui Wang

**Affiliations:** 1 College of Food Science and Engineering, Guiyang University, Guiyang 550005, China; 2 Guizhou Key Laboratory of New Quality Processing and Storage of Ecological Specialty Food, Guiyang 550005, China; 3 College of Liquor and Food Engineering, Guizhou University, Guiyang 550025, China; 4 Academy of Agricultural Planning and Engineering, Ministry of Agriculture and Rural Affairs, Beijing 100125, China; 5 Institute of Agricultural Products Preservation and Processing Technology ( National Engineering and Technology Research Center for Preservation of Agricultural Products), Tianjin Academy of Agricultural Sciences, Tianjin 300384, China; 6 Key Laboratory of Storage and Preservation of Agricultural Products, Ministry of Agriculture and Rural Affairs, Tianjin 300384, China; 7 Tianjin Key Laboratory of Postharvest Physiology and Storage and Preservation of Agricultural Products, Tianjin 300384, China; 8 State Key Laboratory of Vegetable Biobreeding, Tianjin 300384, China

**Keywords:** energy metabolism, ethylene production rate, postharvest management, preservative, shelf-life

## Abstract

The narrow edible window period of kiwifruit poses significant challenges for the high-value fresh fruit industry. This study comparatively evaluated the effects of 1-methylcyclopropene (1-MCP), melatonin (MT), and acetylsalicylic acid (ASA) on “Guichang” kiwifruit by analyzing their physiology, energy metabolism, and transcriptomic profiles. The results indicated that 0.5 μL/L 1-MCP, 1.0 mmol/L MT, and 2.0 mmol/L ASA were the optimal concentrations, all of which significantly inhibited ethylene production and respiration intensity. These treatments effectively maintained fruit firmness, vitamin C content, and high energy status by preserving ATP levels and the activities of H^+^-ATPase and Ca^2+^-ATPase while suppressing the activities of CCO, SDH, 6-PGDH, and G-6-PDH. Transcriptomic analysis and RT-qPCR verification revealed that these preservatives re-programmed key respiratory pathways, including glycolysis, the TCA cycle, the pentose phosphate pathway, and oxidative phosphorylation. MT exhibited transcriptomic responses highly similar to 1-MCP (sharing 13 KEGG pathways) and resulted in the lowest decay rate among treatments, whereas 1-MCP showed the strongest maintenance of core firmness. Our findings suggested that MT exhibited promising performance across multiple quality- and energy-related parameters and may serve as a potential alternative treatment for maintaining kiwifruit quality during the edible window period. Future studies should functionally validate the identified candidate genes and evaluate the effects of MT on sensory and flavor attributes under commercial storage and distribution conditions.

## 1. Introduction

Kiwifruit (*Actinidia* spp.) has gained widespread popularity in the global fruit market owing to its distinctive flavor and rich nutritional value [1,2]. As a typical climacteric fruit, kiwifruit is generally harvested at physiological maturity but remains inedible until a ripening process occurs. This process leads the fruit toward its “edible window period”—a brief phase where the taste and texture are optimal [3]. In general, kiwifruits during the edible window period are defined by a soluble solid content (SSC) ranging from 14% to 21% and a flesh firmness ranging from 10 N to 20 N [4,5].

In practice, applying exogenous ethylene is a common strategy to accelerate the ripening process to reach the edible window period. For instance, Yan et al. [5] found that exogenous ethylene treatment triggers “Guichang” kiwifruit to reach the edible window within 24 h; however, this window is extremely narrow, typically lasting only 3–5 days. The rapid quality deterioration during this narrow window poses a significant challenge for postharvest management and limits the economic benefits of the fruit [6].

To maintain quality and extend this narrow window, the application of fruit preservatives is a commonly utilized strategy [7,8]. Among fruit preservatives, 1-methylcyclopropene (1-MCP) is the most widely adopted agent [9]. As a typical ethylene receptor inhibitor, 1-MCP effectively delays senescence by competitively binding to ethylene receptors [4,10]. Traditionally, 1-MCP is applied before fruits enter the edible window period to prolong storage life and facilitate transport [11,12,13]. Furthermore, a combined treatment of 1-MCP and ethylene has been shown to alleviate chilling injury in kiwifruit [14]. However, recent studies examining the application of 1-MCP to kiwifruit during the edible window period have reported undesirable effects when the treatment is improperly applied, such as hard-core texture and flavor deterioration [15,16]. These findings highlight the need to further optimize 1-MCP application strategies and to explore alternative postharvest preservatives.

Small signaling molecules, such as melatonin (MT) and acetylsalicylic acid (ASA), are emerging as potential fruit preservatives due to their ability to regulate fruit quality via physiological metabolism modulation [17,18,19]. Their efficacy has been demonstrated on other climacteric fruits like tomatoes [20,21], bananas [22], mangoes [23] and muskmelons [24]. As a naturally occurring compound, MT can regulate multiple physiological processes, including lowering respiration intensity [25,26], suppressing ethylene biosynthesis [27], and modulating sugar metabolism during cold storage [28]. Specifically for kiwifruit, recent studies have begun to explore its protective role. For instance, Luo et al. [29] found that exogenous MT treatment can maintain ascorbic acid levels by regulating biosynthesis and regeneration genes in “Jinyan” kiwifruit, thereby delaying fruit senescence. Furthermore, MT application has been shown to alleviate chilling injury in kiwifruit during cold storage [30]. Similarly, ASA, a small phenolic molecule, has also been shown to delay kiwifruit senescence primarily through the modulation of ethylene biosynthesis [31,32]. This regulatory effect is largely mediated by the altered expression of key ethylene-related genes, such as 1-aminocyclopropane-1-carboxylate synthase (*ACS*) and 1-aminocyclopropane-1-carboxylate oxidase (*ACO*) [33]. Although MT and ASA possess clear physiological regulatory functions that are relevant to fruit ripening and senescence, studies exploring their potential to prolong the kiwifruit during edible window period remain limited, and even fewer studies have systematically evaluated the optimal application concentrations and underlying regulatory mechanisms of MT and ASA in kiwifruits during the edible window period.

Therefore, this study aims to optimize the post-ripening preservation of kiwifruit. Specifically, the objectives are to: (1) determine the optimal concentrations of 1-MCP, MT, and ASA for kiwifruits during the edible window period; (2) assess their impacts on fruit physiological quality and senescence parameters; and (3) elucidate their underlying regulatory mechanisms on respiration and energy metabolism during the edible window period.

## 2. Materials and Methods

### 2.1. Fruit Materials

Kiwifruits (*Actinidia chinensis* cv. “Guichang”) were harvested on 26 September 2024, from Xiuwen County (Guizhou Province, China; 106°21′ E, 26°45′ N). A total of 2100 fruits that were uniform in size and without visible defects were selected, with a weight ranging from 70 to 80 g, SSC of 7.78% ± 0.68% and dry matter content of 18.23% ± 1.14%.

The fruits were transported to Guizhou Key Laboratory of New Quality Processing and Storage of Ecological Specialty Food in Guiyang within 2 h after harvest. Upon arrival, the fruits were held at 15 °C ± 3 °C for 24 h to allow for wound healing at the picking scars before ethylene treatment. Subsequently, the kiwifruits were placed in sealed plastic boxes (60 L, 55×40 × 31.5 cm), and ethylene was injected into the boxes to reach a final concentration of 250 μL/L. The O_2_ and CO_2_ concentrations inside the boxes were monitored every 3 h using a headspace analyzer (Check Point II, Dansensor, Ringsted, Denmark). Ripening was terminated when the O_2_ concentration in any group fell below 5% to avoid over-ripening and maintain a uniform physiological stage, following the protocol described in our previous method [5]. After treatment, all kiwifruits reached the pre-edible stage, characterized by a mean SSC of 13.50% ± 0.50%.

### 2.2. Fruit Treatments and Experiment Design

To compare the effects of 1-MCP, MT, and ASA on kiwifruits during the edible window period under low-temperature (4 °C ± 0.5 °C) storage and the subsequent shelf-life period at room temperature (25 °C ± 2.0 °C), two experiments were conducted: (a) screening the optimal concentration of each preservative and (b) comparing the physiological and quality differences in fruits treated with each preservative at its respective optimal concentration. Additional analyses were performed to elucidate the underlying regulatory mechanisms.

In Experiment (a), a total of 900 fruits were randomly allocated to nine treatment levels, consisting of eight preservative concentration treatments and one untreated control (CK). The 1-MCP treatments consisted of 0.25 and 0.5 μL L^−1^ 1-MCP; the MT treatments consisted of 0.1, 0.5, and 1.0 mmol L^−1^ MT; and the ASA treatments consisted of 0.5, 1.0, and 2.0 mmol L^−1^ ASA. Each treatment level consisted of 100 fruits, which were randomly divided into three independent biological replicates containing 33, 33, and 34 fruits, respectively.

For the 1-MCP treatments, the fruits were fumigated with 0.25 or 0.5 μL L^−1^ 1-MCP for 24 h at 25 ± 2 °C. For the MT treatments, the fruits were immersed in the respective MT solutions for 10 min in the dark at 25 ± 2 °C and then air-dried for 24 h. For the ASA treatments, the fruits were immersed in the respective ASA solutions for 5 min at 25 ± 2 °C and then air-dried for 24 h. The CK fruits were maintained under the same conditions at 25 ± 2 °C for 24 h without preservative treatment.

Following treatment, the fruits were stored in open, well-ventilated plastic crates at 4 ± 0.5 °C and 80–90% relative humidity for 5 d, followed by a 5 d shelf-life period at 25 ± 2 °C and 60–70% relative humidity. Assessments were conducted on day 0, immediately after preservative treatment and before cold storage; day 5, at the end of cold storage; and day 10, at the end of the shelf-life period. The evaluated attributes included decay rate, outer pericarp firmness, inner pericarp firmness, core firmness, SSC, respiration intensity, and ethylene production rate. The decay rate was calculated separately for each biological replicate based on the proportion of decayed fruit among the fruit evaluated in that replicate. The sampling and replication used for the other measurements are described in the corresponding analytical sections.

Based on the screening results from Experiment (a), the optimal concentrations were 0.5 μL/L for 1-MCP, 1.0 mmol/L for MT, and 2.0 mmol/L for ASA (see Section 3). These concentrations were therefore selected in Experiment (b). In Experiment (b), a total of 1200 kiwifruits were randomly allocated to three preservative treatment (1-MCP, MT, and ASA) groups and one control group, with 300 fruits per group, and each treatment was divided into three independent biological replicates of 100 fruits. All fruits had been treated with ethylene prior to this experiment to ripen them to the pre-edible stage, characterized by a mean SSC of 13.50% ± 0.50%, consistent with that in Experiment (a).

Subsequently, kiwifruits were treated with the respective preservatives at their optimal concentrations, following the same application procedures as described in Experiment (a). After treatment, the fruits were stored in open, well-ventilated plastic crates at 4 °C ± 0.5 °C for 12 days and then transferred to 25 °C ± 2.0 °C for an additional 11-day shelf-life period.

Physicochemical attributes, including firmness, respiration intensity, and ethylene production rate, were assessed on days 0, 6, 9, and 12 during cold storage and on days 3, 7, and 11 during the shelf-life period. At each sampling time, 18 fruits were collected from each treatment, comprising six fruits from each biological replicate. Fruit appearance, firmness, color, SSC, titratable acidity, vitamin C content, respiration intensity, ethylene production, adenylate contents, energy charge, and the activities of enzymes related to respiratory and energy metabolism were evaluated as described below. For biochemical and molecular analyses, equal amounts of tissue collected from the six fruits within each replicate were pooled to form one biological replicate, resulting in three independently pooled biological replicates for each treatment and sampling time. Tissue samples were immediately frozen in liquid nitrogen and stored at −80 °C until analysis. Transcriptomic analysis was performed using samples collected on day 0 and from the CK, 1-MCP, MT, and ASA groups on shelf-life day 11.

### 2.3. Determination of Fruit Physical Quality Attributes

The decay rate was determined by calculating the percentage of decayed fruits within each treatment and control group [34].

Fruit firmness (18 fruits in total, comprising six fruits from each of the three biological replicates) was measured using a texture analyzer (EZ-Test, Shimadzu Corporation, Kyoto, Japan). Measurements were conducted at three specific positions of each fruit: the outer pericarp, inner pericarp, and core. The outer and inner pericarp firmness was determined using a 2.0 mm diameter probe, while a 4.0 mm diameter probe was employed for core firmness. Firmness values were expressed as the maximum penetration force in Newtons (N).

Fruit peel and pulp color were determined using a colorimeter (CR-410, Konica Minolta, Inc., Tokyo. Japan). For each treatment and sampling time, 18 fruits were measured, comprising six fruits from each biological replicate. Color parameters were expressed as L*, a* and b*. The hue angle (H°) of the fruit peel was calculated according to the following equation:

$$H^{{}^{\circ}}=\tan^{-1}\left(\frac{b^{*}}{a^{*}}\right)$$

### 2.4. Determination of SSC, Titratable Acid (TA), SSC/TA Ratio and Vitamin C (Vc) Content

The SSC (9 fruits in total, comprising three fruits from each of the three biological replicates) was measured using a digital refractometer (PAL-1, ATAGO, Tokyo, Japan) and expressed as a percentage. TA (9 fruits in total, comprising three fruits from each of the three biological replicates) was determined by titrating the extracted juice with 0.1 mol/L NaOH to an endpoint of pH 8.3, and the results were expressed as a percentage of citric acid [35]. The SSC/TA ratio was calculated as the SSC divided by TA. For vitamin C (Vc) determination (9 fruits in total, comprising three fruits from each of the three biological replicates), pooled pulp tissue was immediately frozen in liquid nitrogen and stored at −80 °C until analysis. The frozen tissue was homogenized, and Vc content was determined by a spectrophotometric iron reduction assay, and the results were expressed as mg/100 g of fresh weight [36].

### 2.5. Determination of Respiration Intensity and Ethylene Production Rate

Respiration intensity and the ethylene production rate were measured using 18 fruits, comprising six fruits from each of the three biological replicates. For each replicate, six fruits were sealed in a 1.0 L airtight jar for 4 h at 25 °C ± 2.0 °C. The CO_2_ concentration was measured using an infrared gas analyzer (Check Point 3; Dansensor, Denmark), and respiration intensity was expressed as mg CO_2_ kg^−1^ h^−1^. Subsequently, a 2 mL gas sample was extracted from the headspace and injected into a gas chromatograph (GC-2014C, Shimadzu, Tokyo, Japan) to determine the ethylene production rate, following the method of Zhang et al. [37]. The operating conditions were: column temperature 80 °C, injector temperature 145 °C, and detector temperature 180 °C. The results were expressed in µg kg^−1^ h^−1^.

### 2.6. Determination of Adenosine Phosphate and Energy Charge (EC)

For each treatment and sampling time, pulp tissues collected from the six fruits within each biological replicate were pooled to form one composite sample. Three independent biological replicates were analyzed. The pooled tissue samples were immediately frozen in liquid nitrogen and stored at −80 °C until analysis.

The levels of adenosine triphosphate (ATP), adenosine diphosphate (ADP), and adenosine monophosphate (AMP) were quantified via high-performance liquid chromatography (HPLC) according to Zhang et al. [38]. Analysis was performed using an UltiMate 3000 HPLC system (Thermo Fisher Scientific, Shanghai, China) equipped with a UV detector and an Agilent Zorbax SB-C18 column (250 mm × 4.6 mm, 5 µm; Agilent Technologies Inc., Santa Clara, CA, USA). Adenylate concentrations were expressed as µg g^−1^ on a fresh weight basis. The energy charge (EC) was calculated using the following equation:

$$EC=\frac{\left[ATP]+\frac{1}{2}[ADP\right]}{\left[ATP]+[ADP]+[AMP\right]}$$

### 2.7. Determination of Respiratory and Energy Metabolism-Related Enzyme Activities

For each treatment and sampling time, equal amounts of pulp tissue collected from the six fruits within each biological replicate were pooled to form one composite sample. Three independent biological replicates were analyzed. The pooled tissue samples were immediately frozen in liquid nitrogen and stored at −80 °C until analysis.

Frozen pulp tissue (2 g) stored at −80 °C was used for enzymatic extraction and quantification. The activities of key enzymes involved in respiratory pathways, including succinate dehydrogenase (SDH), cytochrome c oxidase (CCO), glucose-6-phosphate dehydrogenase (G-6-PDH), and 6-phosphogluconate dehydrogenase (6-PGDH), as well as the activities of membrane-bound energy-utilizing proteins (H^+^-ATPase and Ca^2+^-ATPase), were determined using commercial assay kits (Suzhou Comin Biotechnology Co., Ltd., Suzhou, China).

### 2.8. RNA Extraction, Library Construction, and Transcriptomic Sequencing

Transcriptomic analysis was conducted using samples collected on day 0 and from the CK, ASA, MT, and 1-MCP groups on shelf-life day 11. For each treatment or sampling stage, pulp tissues collected from six fruits within each biological replicate were pooled to form one composite sample. Three independent biological replicates were used, resulting in a total of 15 samples for transcriptomic sequencing. All tissue samples were immediately frozen in liquid nitrogen and stored at −80 °C until RNA extraction.

Total RNA was extracted from frozen kiwifruit tissue stored at −80 °C using a magnetic tissue total RNA kit (Tiangen Biotech Co., Ltd., Beijing, China). RNA concentration and purity were evaluated using a NanoDrop 2000 spectrophotometer (Thermo Scientific, Waltham, MA, USA), while RNA integrity was verified using an Agilent 2100 Bioanalyzer (Agilent Technologies, Santa Clara, CA, USA).

For library construction, mRNA was enriched using oligo (dT) magnetic beads and subsequently fragmented. First- and second-strand cDNA were synthesized, followed by end-repair, A-tailing, and adapter ligation. The resulting libraries were amplified by PCR and purified. Library quality was assessed using an Agilent 2100 Bioanalyzer in conjunction with an Agilent High Sensitivity DNA Kit (Agilent Technologies) to ensure appropriate insert size and library purity. The library concentration was measured using PicoGreen (Quantifluor-ST fluorometer, Promega, Madison, WI, USA) and quantified by qPCR (StepOnePlus Real-Time PCR System, Thermo Scientific). The libraries were homogenized, diluted and sequenced in PE150 mode on an Illumina sequencer (Shanghai Baipu Biotechnology Co., Ltd. Shanghai, China).

### 2.9. RNA-Seq Analysis and RT-qPCR Validation

The raw sequencing reads were filtered to remove adapter sequences and reads with an average quality score lower than Q20, and the resulting clean reads were used for further analysis. The “Hongyang” genome (version 3.0, https://kiwifruitgenome.org/organism/5, accessed on January 2025) was used as the reference genome. HISAT2 (v2.1.0) was used to construct the reference genome index and align paired-end reads.

Differentially expressed genes (DEGs) were identified using DESeq, with the selection criteria of |log_2_ (fold change)| > 1 and adjusted *p*-value < 0.05. Gene Ontology (GO) and Kyoto Encyclopedia of Genes and Genomes (KEGG) enrichment analyses were conducted to elucidate the biological functions and pathways associated with DEGs in kiwifruits.

For the validation of transcriptomic profiles, six representative genes associated with energy and respiratory metabolism were subjected to RT-qPCR. The relative mRNA abundance was calculated via the 2^−ΔΔCt^ method and normalized to the *Actin* internal control [37]. Specific primers (Table S1) were designed and supplied by Bio-Transduction Lab Co., Ltd. (Wuhan, China).

### 2.10. Statistical Analysis

This experiment was conducted using three independent biological replicates for each treatment. For measurements involving multiple fruits within each biological replicate, the measurements obtained from the individual fruits were averaged to generate one replicate value. The decay rate was calculated separately for each biological replicate based on the proportion of decayed fruit among the fruit evaluated in that replicate. Data are presented as the mean ± standard deviation (SD) of three independent biological replicates.

Before analysis, the normality and homogeneity of variance of the data were assessed using the Shapiro–Wilk test and Levene’s test, respectively. All datasets satisfied the assumptions of normality and homogeneity of variance. At each sampling time, differences among treatments were evaluated using a one-way analysis of variance (ANOVA). Duncan’s multiple range test was used to evaluate statistical significance (*p* < 0.05). Differences were considered statistically significant at *p* < 0.05. Statistical analyses were performed using SPSS Statistics 27.0 (IBM Corp., Armonk, NY, USA), and figures were prepared using GraphPad Prism 10.1.2 (GraphPad Software, Boston, MA, USA).

Principal component analysis (PCA) was performed using the normalized gene expression matrix to evaluate overall transcriptomic variation and the reproducibility of biological replicates.

## 3. Results

### 3.1. Effects of 1-MCP, MT, and ASA Concentrations on Edible Window Period

Among the tested concentrations, 0.5 μL/L 1-MCP (Figure S1), 1.0 mmol/L MT (Figure S2), and 2.0 mmol/L ASA (Figure S3) most effectively suppressed decay, respiration intensity, and ethylene production while delaying firmness loss. These concentrations were selected as the optimal levels for subsequent experiments.

### 3.2. Effects on Fruit Quality During the Edible Window Period

**Figure 1:**
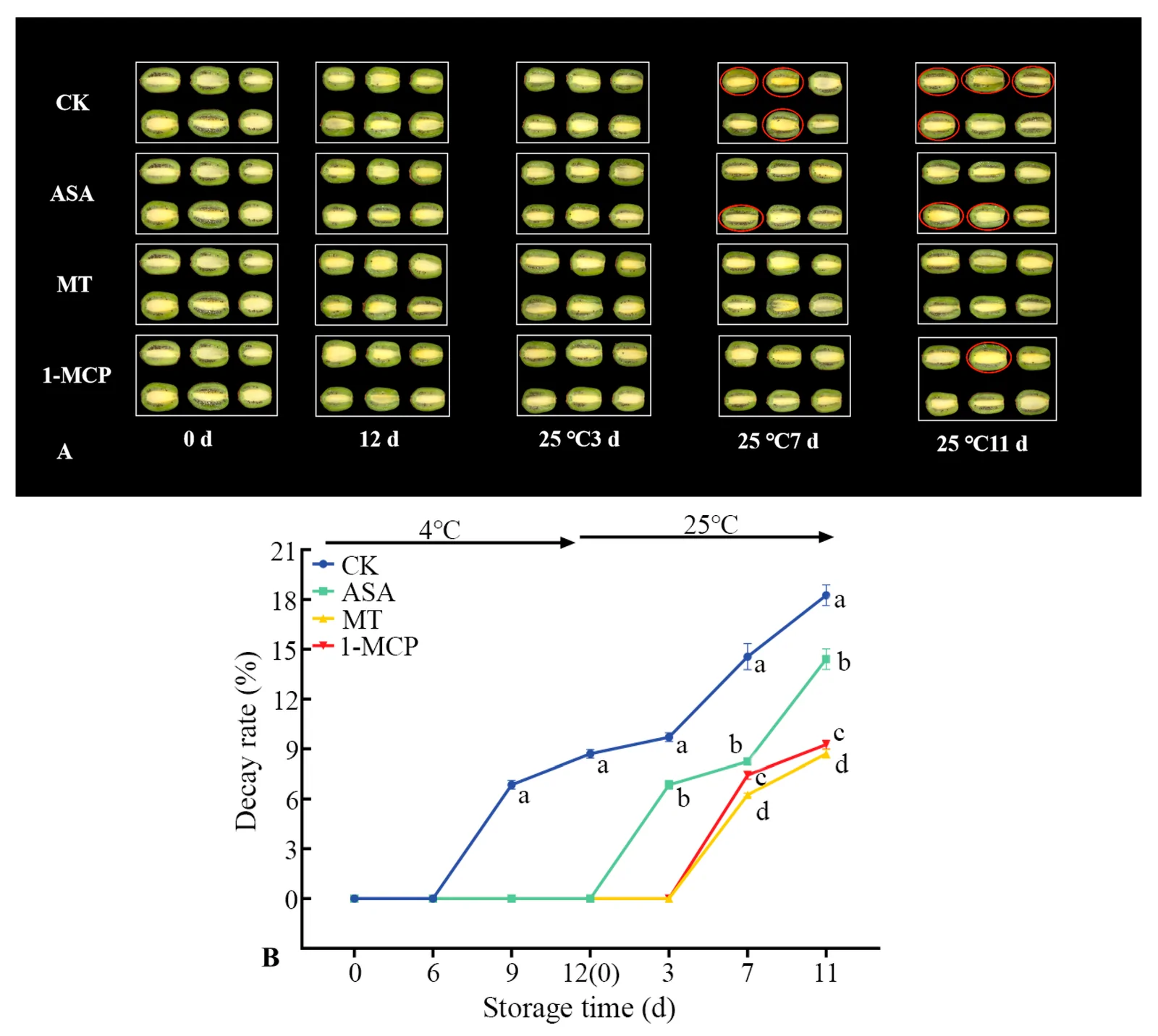
Changes in appearance (**A**) and decay rate (**B**) during cold storage at 4 ± 0.5 °C and subsequent shelf-life at 25 ± 2 °C. In (**A**), Red circles indicate decayed fruit. In (**B**), data are presented as mean ± SD (n = 3 biological replicates), and different letters indicate significant differences between treatments at each time point according to Duncan’s multiple range test (*p* < 0.05). Absence of letters at given time point indicates no significant difference among treatments.

No visible browning was observed in any group during cold storage. Browning appeared on day 7 of shelf-life in the CK and ASA groups and intensified by day 11, whereas only slight browning was observed in the 1-MCP treatment on day 11, and the MT treatment uniquely maintained the fruit’s initial appearance throughout the entire experimental period (Figure 1A).

The decay rate increased progressively in all groups during shelf-life. All treatments (1-MCP, MT, and ASA) significantly reduced decay compared with the CK group (*p* < 0.05), with MT showing the strongest effect. By day 11, decay rates in the ASA, 1-MCP, and MT groups were reduced by 20.78%, 49.09%, and 52.17%, respectively, relative to CK (Figure 1B).

**Figure 2:**
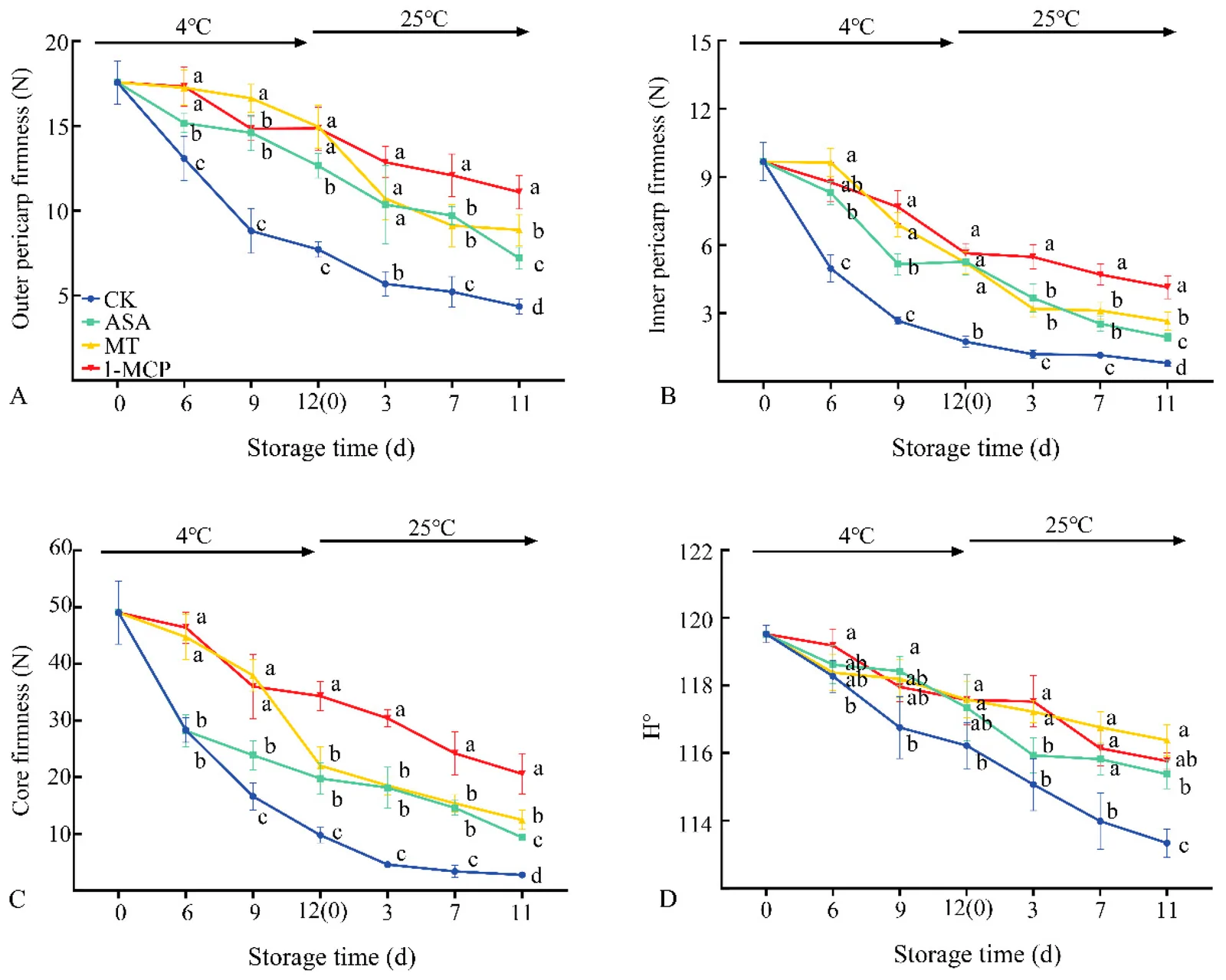
Variations in firmness and pulp color of kiwifruit during edible window period. (**A**) Outer pericarp firmness. (**B**) Inner pericarp firmness. (**C**) Core firmness. (**D**) Pulp hue angle (H°). Data are presented as mean ± SD (n = 3 biological replicates). Different letters indicate significant differences between treatments at each time point according to Duncan’s multiple range test (*p* < 0.05). Absence of letters at given time point indicates no significant difference among treatments.

The firmness of the outer pericarp, inner pericarp, and core tissues declined during storage (Figure 2A–C), with the fastest softening observed in the CK group. During cold storage, firmness decreased rapidly in CK fruit, whereas all treatments slowed the rate of decline. After transfer to shelf-life conditions, softening was further accelerated in CK fruit, while treated fruit maintained substantially higher firmness. Differences among treatments became increasingly pronounced during shelf-life, particularly in the core tissue, where 1-MCP consistently showed the strongest effect, followed by MT and ASA. By day 11, core firmness in the 1-MCP group (22.31 ± 1.48 N) remained markedly higher than in the other groups (*p* < 0.05).

The pulp hue angle (H°) decreased progressively during storage in all groups (Figure 2D), with a faster decline in CK fruit. All treatments slowed the decrease in H°, and higher values were maintained in the 1-MCP and MT groups compared with ASA during shelf-life.

**Figure 3:**
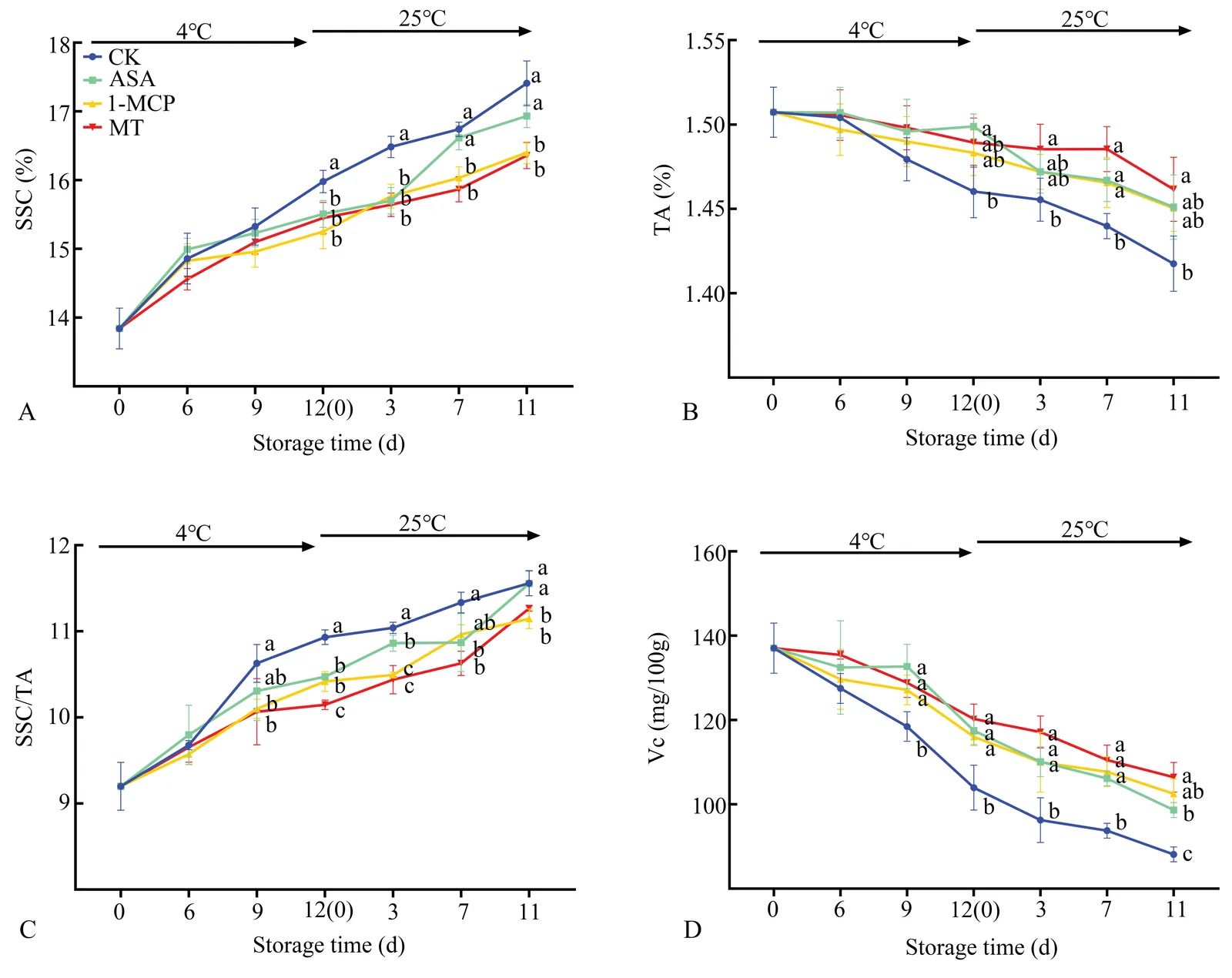
Internal quality of kiwifruit during edible window period under various treatments. (**A**) Soluble solid content (SSC). (**B**) Titratable acidity (TA). (**C**) SSC/TA ratio. (**D**) Vitamin C (Vc) content. Data are presented as mean ± SD (n = 3 biological replicates). Different letters indicate significant differences between treatments at each time point according to Duncan’s multiple range test (*p* < 0.05). Absence of letters at given time point indicates no significant difference among treatments.

SSC increased in all groups during storage, whereas TA and Vc decreased (Figure 3A,B,D). Compared with CK, MT and 1-MCP significantly suppressed the increase in SSC and maintained higher TA and Vc levels during shelf-life (*p* < 0.05), while ASA showed a weaker effect. Consequently, the SSC/TA ratio increased in all groups but remained lower in MT- and 1-MCP-treated fruit (Figure 3C).

### 3.3. Effects on the Ethylene Production Rate, Respiration Intensity, and Energy Status During the Edible Window Period

**Figure 4:**
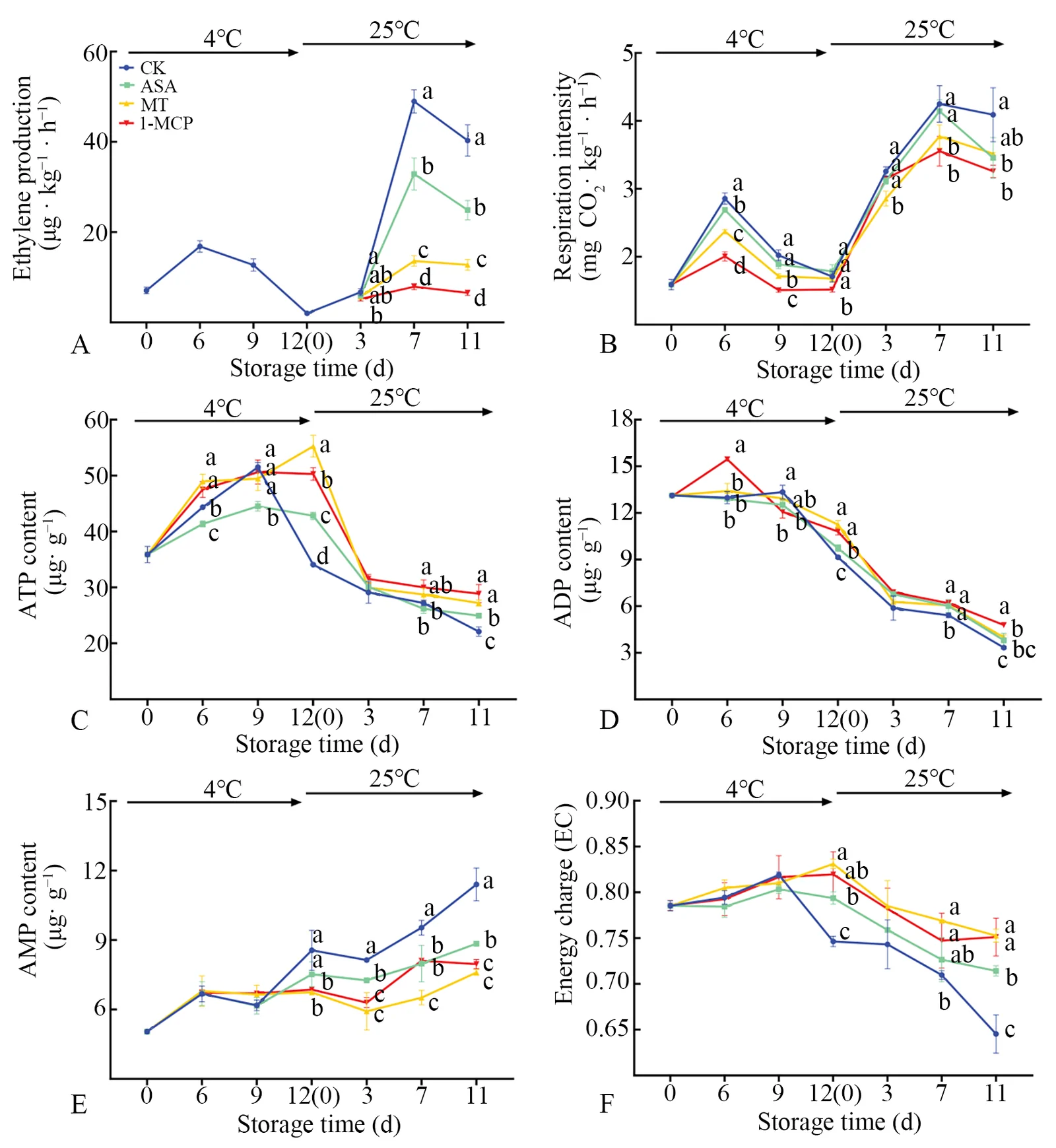
Ethylene production rate, respiration intensity, and energy status of kiwifruit during edible window period. (**A**) Ethylene production rate. (**B**) Respiration intensity. (**C**) ATP content. (**D**) ADP content. (**E**) AMP content. (**F**) Energy charge (EC). Data are presented as mean ± SD (n = 3 biological replicates). Different letters indicate significant differences between treatments at each time point according to Duncan’s multiple range test (*p* < 0.05). Absence of letters at given time point indicates no significant difference among treatments.

Ethylene production and respiration intensity followed a climacteric pattern in all groups (Figure 4A,B). During cold storage, ethylene production peaked on day 6 in the CK group, whereas no ethylene production was detected in the 1-MCP-, MT-, or ASA-treated groups. During shelf-life, ethylene production reached a peak on day 7 in all groups; however, the peak values were significantly lower in treated fruit than in the CK group (*p* < 0.05). Similarly, the respiration intensity of all groups followed a peak-and-decline pattern during cold storage, reaching a maximum on day 6 (Figure 4B). At this peak, the respiration intensity in the CK group was 1.06-, 1.21-, and 1.43-fold higher than those in the ASA, MT, and 1-MCP groups, respectively (*p* < 0.05).

ATP levels initially increased and then declined during storage, while ADP decreased, and AMP increased (Figure 4C–E). Treated fruit maintained significantly higher ATP levels and energy charge (EC) than CK during shelf-life (*p* < 0.05), particularly under the MT and 1-MCP treatments (Figure 4C,F).

### 3.4. Effects on Enzymatic Regulation of Energy Metabolism During Edible Window Period

**Figure 5:**
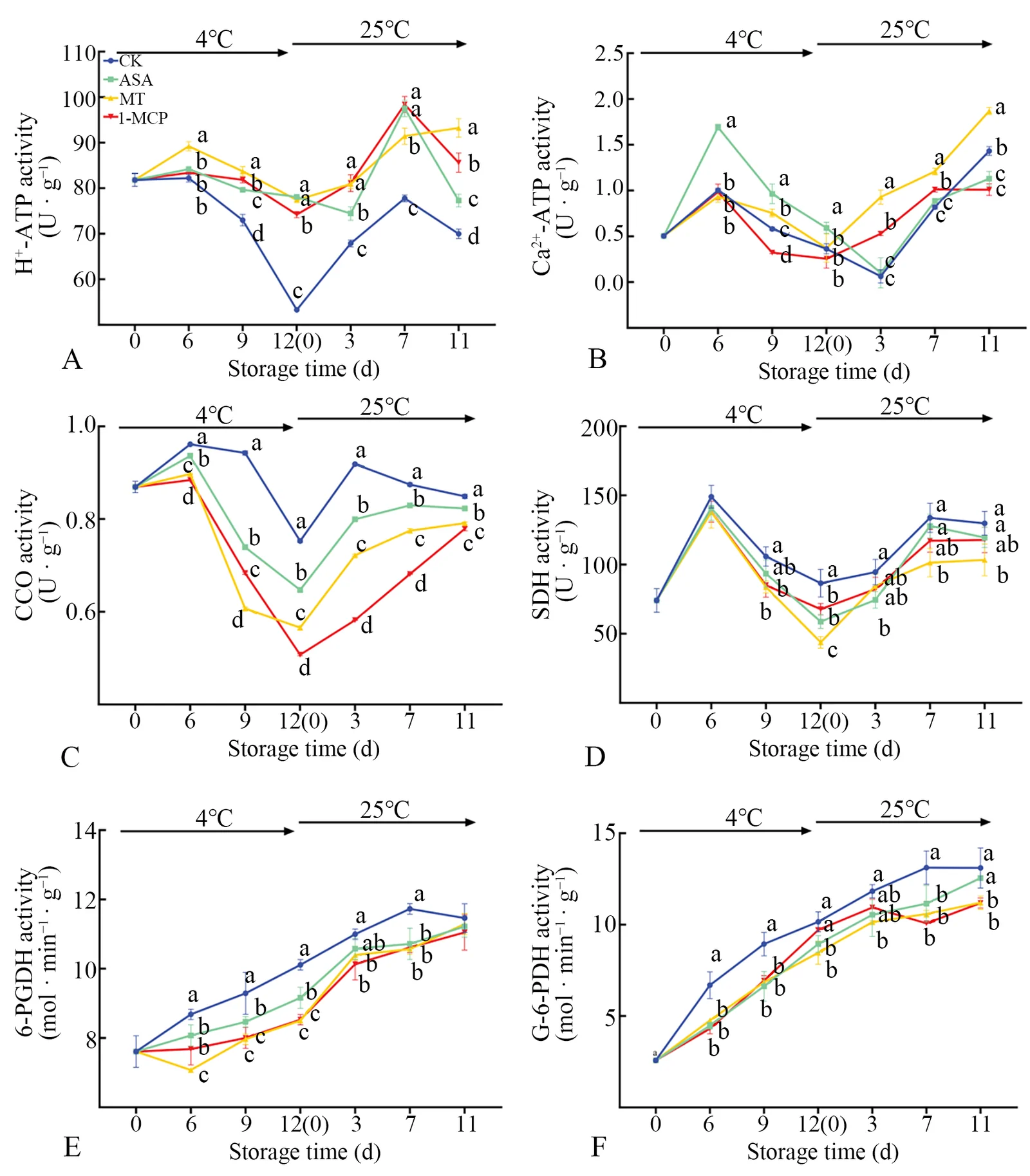
Enzyme activities associated with energy metabolism of kiwifruit during edible window period. (**A**) H^+^-ATPase activity. (**B**) Ca^2+^-ATPase activity. (**C**) CCO activity. (**D**) SDH activity. (**E**) Glucose-6-phosphate dehydrogenase (G-6-PDH) activity. (**F**) 6-phosphogluconate dehydrogenase (6-PGDH) activity. Data are presented as mean ± SD (n = 3 biological replicates). Different letters indicate significant differences between treatments at each time point according to Duncan’s multiple range test (*p* < 0.05). Absence of letters at given time point indicates no significant difference among treatments.

H^+^-ATPase activity initially increased and then decreased both during cold storage and shelf-life (Figure 5A). From day 9 of cold storage to day 11 of the shelf period, the H^+^-ATPase activities in the ASA, MT and 1-MCP groups were significantly higher than that in the CK group (*p* < 0.05). A similar trend was observed for Ca^2+^-ATPase (Figure 5B). During the shelf-life period, the MT group exhibited significantly higher Ca^2+^-ATPase activity compared with other treatment groups (*p* < 0.05).

CCO and SDH, as key enzymes in the mitochondrial respiratory chain, exhibited lower activities in the ASA, MT and 1-MCP groups compared with the CK group (Figure 5C,D), which was consistent with the observed respiration intensity (Figure 4B), indicating that the treatments effectively suppressed excessive respiratory metabolism. Glucose-6-phosphate dehydrogenase (G-6-PDH) and 6-phosphogluconate dehydrogenase (6-PGDH), which are essential for NADPH production in the pentose phosphate pathway, were monitored (Figure 5E,F). The activities of G-6-PDH and 6-PGDH in the treatment groups remained consistently lower throughout the storage period, whereas they were significantly higher in the CK group (*p* < 0.05).

### 3.5. Global Transcriptomic Analysis of Kiwifruit During Edible Window Period

**Figure 6:**
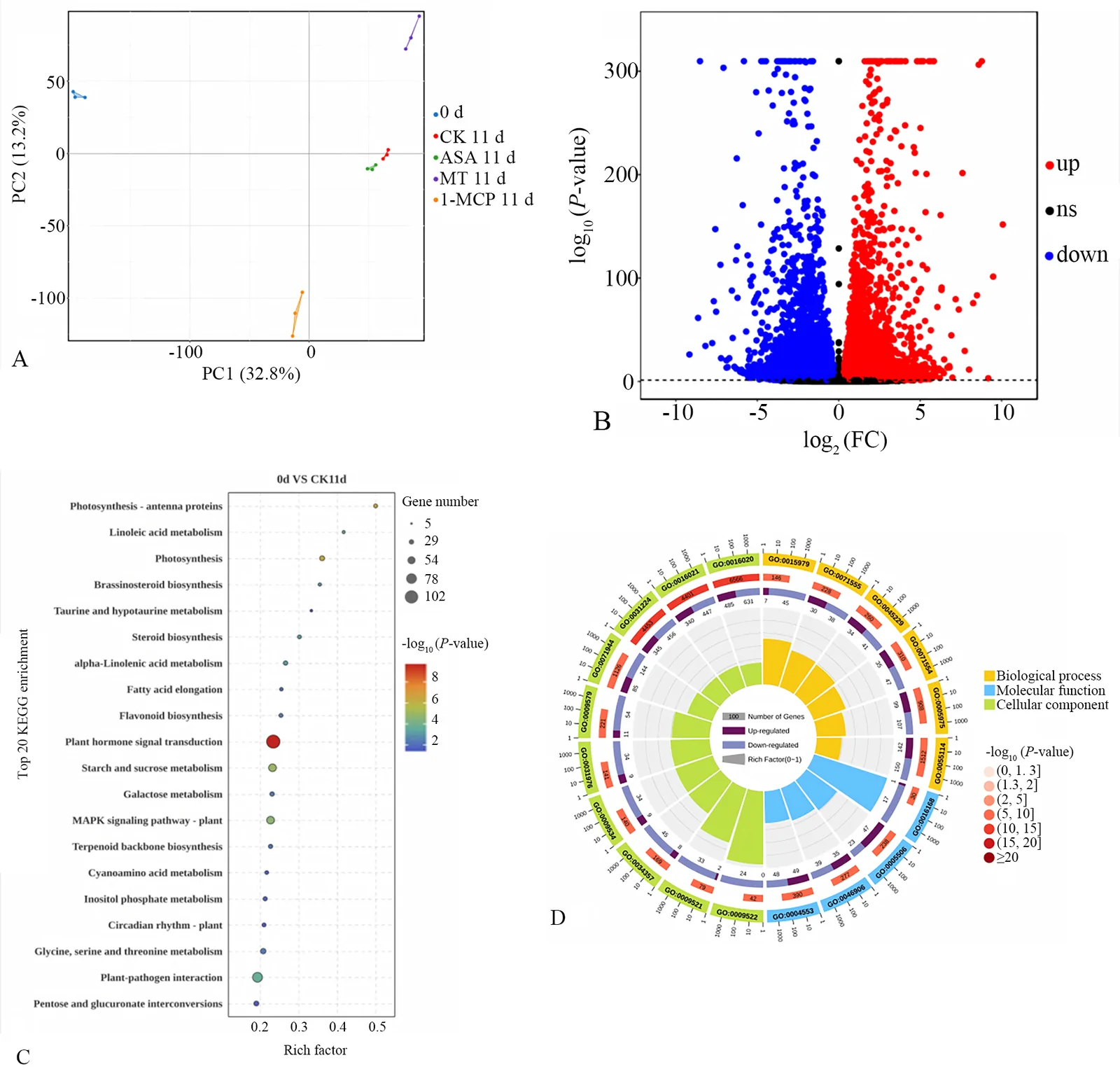
Transcriptomic analysis of differentially expressed genes (DEGs) in kiwifruit during edible window period. (**A**) Principal component analysis (PCA) of transcriptomic data across all samples (0 d, CK 11 d, ASA 11 d, MT 11 d, and 1-MCP 11 d). (**B**) Volcano plot of DEGs in comparison of 0 d vs. CK 11 d. (**C**) Top 20 significantly enriched KEGG pathways for DEGs between 0 d and 11 d in CK group. (**D**) Top 20 enriched GO terms (Biological Process, Molecular Function, and Cellular Component) for DEGs between 0 d and 11 d in CK group.

Transcriptome sequencing yielded high-quality data, with PCA clearly separating samples according to storage time and treatment (Figure 6A). The first two principal components explained 32.8% and 13.2% of the total variance, respectively, and biological replicates clustered tightly, indicating high reproducibility.

A total of 3751 DEGs were identified between 0 d and CK at 11 d, including 1965 up-regulated and 1786 down-regulated genes (Figure 6B). KEGG enrichment analysis showed that these DEGs were mainly involved in plant hormone signal transduction, carbohydrate metabolism, and MAPK signaling pathways, while GO analysis highlighted enrichment in membrane components and hydrolase activity, particularly those acting on glycosyl bonds (Figure 6C,D).

**Figure 7:**
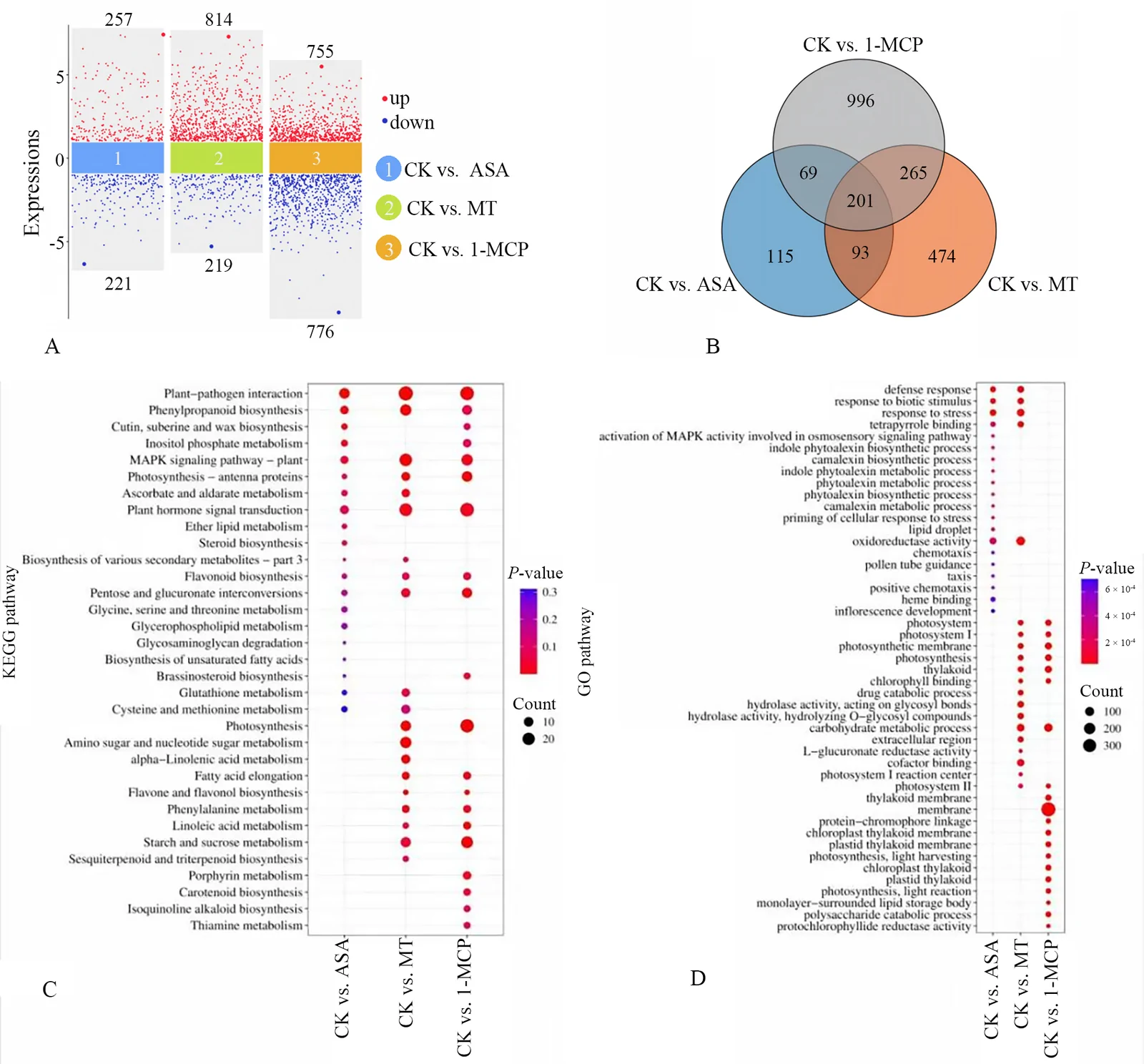
Comparison of differentially expressed genes (DEGs) among treatments on day 11 of the shelf-life period. All comparisons in panels A–D involve samples collected on day 11d. (**A**) Numbers of upregulated and downregulated DEGs in the CK vs. ASA, CK vs. MT, and CK vs. 1-MCP comparisons. (**B**) Venn diagram showing the overlap of DEGs among these three comparisons. (**C**) Top 20 enriched KEGG pathways in each comparison. (**D**) Top 20 enriched GO terms in each comparison.

Compared with CK, all treatments induced distinct transcriptional responses, with 1-MCP showing the largest number of DEGs (1531), followed by MT and ASA (Figure 7A). A core set of 201 DEGs was shared among all treatments (Figure 7B), suggesting partially overlapping regulatory mechanisms. Enrichment analysis revealed both shared and treatment-specific pathways among treatments (Figure 7C,D).

**Figure 8:**
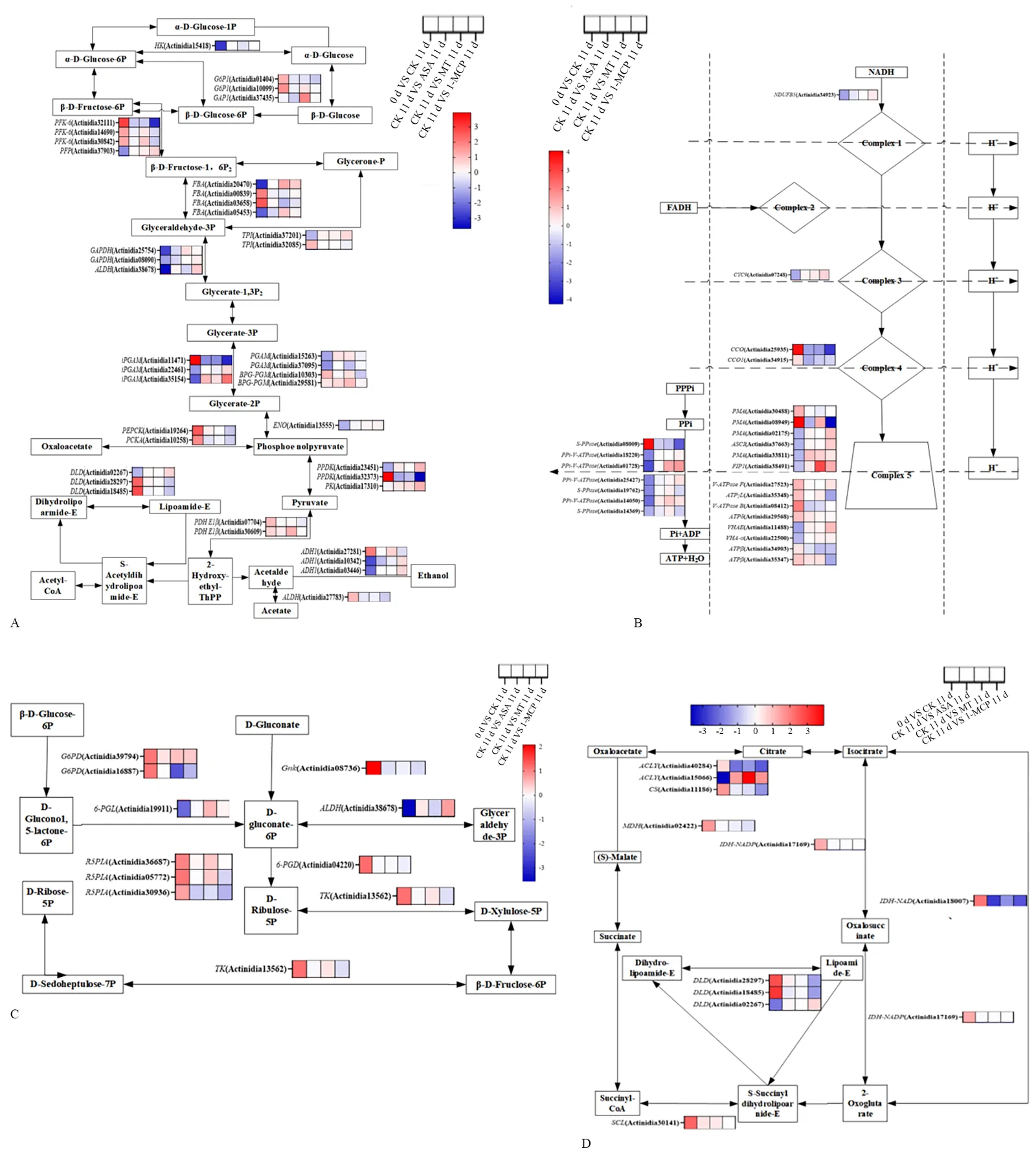
Differentially expressed genes (DEGs) involved in core respiratory metabolism pathways. (**A**) Glycolysis and gluconeogenesis. (**B**) Oxidative phosphorylation. (**C**) Pentose phosphate pathway (PPP). (**D**) Tricarboxylic acid (TCA) cycle. Heatmaps illustrate relative expression levels (|log_2_ (fold change)| > 1) of key genes in four comparisons: 0 d vs. CK 11 d, CK 11 d vs. ASA 11 d, CK 11 d vs. MT 11 d, and CK 11 d vs. 1-MCP 11 d. Red and blue boxes represent up-regulated and down-regulated genes, respectively.

Notably, pathways related to energy metabolism, including glycolysis, oxidative phosphorylation, the pentose phosphate pathway, and the TCA cycle, were differentially regulated during storage (Figure 8). In glycolysis, several genes such as *PFK* and *PDH E1β* were up-regulated during ripening, while treatments modulated their expression. The gene *iPGAM-Actinidia11471* was consistently down-regulated across all treatments. In the oxidative phosphorylation pathway, *CCO-Actinidia25935* was also consistently down-regulated. In the PPP and TCA cycle, key enzymes including G-6-PDH and ACLY showed differential regulation between CK and treated groups.

## 4. Discussion

### 4.1. Concentration Screening and Its Impact on Ethylene-Mediated Ripening

Kiwifruit is a typical climacteric fruit, and its ripening and senescence after harvest are mainly controlled by an integrated regulatory network of ethylene and respiration. Currently, 1-MCP is the most popular preservative for kiwifruit [9]; however, many studies and practical applications have shown that improper 1-MCP treatment can cause “hard-core” issues and flavor loss [11,12,13]. Therefore, finding alternative preservatives and determining their best concentrations is a key goal of our study. Through preliminary experiments, we found that 0.5 μL/L 1-MCP, 1.0 mmol/L MT, and 2.0 mmol/L ASA were the most suitable concentrations (Figures S1–S3). These treatments not only reduced the decay rate but also protected the flavor and nutritional quality of “Guichang” kiwifruit during the edible window period.

Since elevated respiration intensity and accelerated ethylene production rates work synergistically to deplete internal nutrients, they collectively drive the deterioration and senescence of postharvest fruit [39,40]. Consequently, the good performance of these treatments is closely related to their ability to effectively modulate these two key physiological processes (Figure 4A,B). The patterns of ethylene production differed markedly among treatments. While CK fruit showed a typical climacteric peak and ASA-treated fruit still exhibited a noticeable increase in ethylene production during shelf-life, the 1-MCP and MT groups displayed much smoother fluctuation patterns without a pronounced peak. The strong and sustained suppression observed in 1-MCP-treated fruit is likely related to 1-MCP’s established role as an ethylene receptor antagonist, which blocks ethylene perception and thereby suppresses autocatalytic ethylene biosynthesis [10]. In contrast, the relatively stable ethylene production in MT-treated fruit may be associated with MT’s regulatory effects on ethylene biosynthesis [41], particularly through the modulation of key enzymes such as ACC synthase (ACS) and ACC oxidase (ACO) [6]. ASA also reduced ethylene production compared with CK, but its retention of a climacteric-like peak suggests that its inhibitory effect was weaker than that of 1-MCP and MT. This may indicate that ASA alleviated ripening-associated ethylene accumulation to some extent, possibly through crosstalk with salicylic acid-related signaling [42], but did not fully suppress the climacteric response. However, these mechanistic interpretations were not directly examined in the present study and require further validation. Because the ripening process was slowed down, the physical quality of the fruit was better maintained, such as the delayed reduction in firmness and Vc content (Figure 3B,D).

The delayed changes in SSC, TA, vitamin C content, and pulp hue angle further indicate that MT and 1-MCP slowed the overall ripening process more effectively than ASA. The lower SSC/TA ratios suggest a slower shift in sugar–acid balance, while the maintenance of Vc and pulp hue angle reflects reduced nutritional and color deterioration. These responses were generally consistent with the firmness and decay results, although the relative advantages of MT and 1-MCP varied among quality attributes: MT resulted in the lowest decay rate, whereas 1-MCP maintained the highest core firmness. Future studies combining these physicochemical measurements with sensory evaluation and volatile profiling would enable a more comprehensive assessment of treatment effects on the edible quality and flavor of kiwifruit.

### 4.2. Maintenance of Energy Homeostasis Is Essential for Delaying Kiwifruit Senescence

ATP plays a pivotal role in cellular metabolism, and its depletion is a major factor that triggers postharvest fruit senescence [43]. In general, climacteric fruit undergoes rapid ripening during the edible window period, which requires a large amount of energy for various biochemical reactions [44]. In our study, the CK group showed a quick decrease in ATP levels and energy charge (EC), which occurred alongside the rapid decline in fruit quality (Figure 4C,F). However, the 1-MCP, MT, and ASA treatments significantly maintained higher ATP and ADP levels while delaying the increase in AMP (Figure 4C–E). These results are consistent with previous findings that exogenous 1-MCP and MT can effectively preserve the energy status of fruits during storage [29,45,46]. The higher energy levels in the treated fruit were supported by the activities of energy-related enzymes. H^+^-ATPase and Ca^2+^-ATPase are pivotal enzymes for energy conversion, and they are essential for maintaining cellular ion balance and function [47]. We observed that the activities of these two enzymes were significantly higher in all treatment groups compared to the CK group (Figure 5A,B). This maintenance of ATPase activity is critical because it ensures the stability of the mitochondrial membrane and the cell–matrix [48,49]. Among the three treatments, MT showed a slightly better ability to maintain these enzyme activities, which may explain why the MT-treated group exhibited the lowest decay rate during the edible window period (Figure 1B). By keeping a higher energy status and a lower respiration intensity, the treatment groups were able to provide the necessary energy to support defense responses and maintain the integrity of cell membranes, thereby extending the shelf-life of the fruit.

### 4.3. Transcriptomic Regulation of Respiratory and Metabolic Pathways

To further understand the molecular basis of the physiological changes, we analyzed the DEGs involved in major respiratory pathways. Respiration provides the carbon skeletons and energy needed for fruit ripening, but excessive respiration leads to nutrient depletion and early senescence [50]. Our transcriptomic data indicated that genes involved in glycolysis, the TCA cycle, and oxidative phosphorylation showed transcriptional adjustments in response to the ASA, MT, and 1-MCP treatments, which were consistent with the observed changes in energy metabolism.

In the glycolysis pathway, the 1-MCP and MT treatments up-regulated genes such as *GAP1* and *PK*, which might help maintain a more efficient glycolytic flow to provide enough pyruvate for ATP production. This molecular evidence supports our observation that treated fruits maintained higher ATP and ADP levels. Furthermore, the oxidative phosphorylation pathway, which is the final stage of energy production, showed interesting changes. We found that *CCO-Actinidia25935* was consistently down-regulated by all three treatments. Since CCO is a key enzyme in the electron transport chain [51], its suppression likely helps the fruit avoid the excessive energy loss associated with the respiratory climacteric.

Another important finding is related to the pentose phosphate pathway (PPP). In the CK group, we saw an increase in 6-PGDH and G-6-PDH activities and the up-regulation of *6-PGDH*, suggesting that the fruit was activating its antioxidant defenses to fight senescence (Figure 5E,F and Figure 8C). Interestingly, the 1-MCP and MT treatments did not activate the PPP genes as strongly as the CK group did. We speculate that because these treatments effectively maintained fruit quality, the cellular environment remained more stable, and the reactive oxygen species (ROS) balance was not severely disrupted. Therefore, the fruit did not need to trigger the PPP defense mechanism as early as the CK group.

To ensure the reliability of these transcriptomic results, we selected six representative DEGs, including *PFK6*, *iPGAM*, and *CCO*, for RT-qPCR validation. The expression patterns of these genes were highly consistent with the RNA-Seq data (Supplementary Figure S4). For instance, the expression of *PFK6* and *CCO* was significantly lower in the treatment groups than in the CK group, which matches our findings on respiration intensity and enzyme activities. This consistency confirms that our molecular analysis is robust and accurately reflects the metabolic changes in edible-window-period kiwifruit.

## 5. Conclusions

In conclusion, this study identified 0.5 μL/L 1-MCP, 1.0 mmol/L MT, and 2.0 mmol/L ASA as the optimal concentrations for extending the shelf-life of kiwifruit during its edible window period. All three preservatives effectively delayed fruit senescence and maintained quality by suppressing the respiration intensity and sustaining a high-energy status. Notably, MT exhibited the most pronounced efficacy in retarding the escalation in the decay rate. While 1-MCP and MT showed comparable effects in maintaining ATP, EC, and nutritional components (Vc, TA, and SSC), MT outperformed 1-MCP in preserving higher H^+^-ATPase and Ca^2+^-ATPase activities, suggesting its superior capacity for bioenergetic regulation.

Transcriptomic analysis revealed that these preservatives prolonged fruit shelf-life by re-programming key pathways, including glycolysis/gluconeogenesis, the TCA cycle, the PPP, and oxidative phosphorylation. The expression patterns of *iPGAM* (*Actinidia11471*), *CCO* (*Actinidia25935*), and *PFK6* (*Actinidia32111*) suggest that these genes are potential candidates associated with cellular energy homeostasis. Furthermore, the high degree of similarity in KEGG pathways and DEGs between the 1-MCP and MT groups—superior to that of the ASA group—further substantiates the potent preservation potential of MT. Overall, MT showed promising performance as a potential alternative treatment for postharvest kiwifruit, although its relative advantages over 1-MCP varied among the evaluated attributes. Further studies integrating functional gene validation, sensory evaluation, and volatile profiling under commercial storage and distribution conditions are needed to confirm the underlying mechanisms and comprehensively assess the effects of MT on edible quality.

## Data Availability

The original contributions presented in this study are included in the article/Supplementary Materials. Further inquiries can be directed to the corresponding authors.

## Supplementary Materials

The following supporting information can be downloaded at: https://www.mdpi.com/article/10.3390/foods15193489/s1, Table S1. Primers used for RT-qPCR analysis of gene expression in this study. Table S2. Overview of transcriptome sequencing quality for 15 kiwifruit libraries across different treatment groups during the edible window period. Figure S1. Effects of 1-methylcyclopropene (1-MCP) treatment at different concentrations (0.25 and 0.5 μL/L) on the physiological quality of kiwifruit during the edible window period. Figure S2. Effects of melatonin (MT) treatment at different concentrations (0.1, 0.5, and 1.0 mmol/L) on the physiological quality of kiwifruit during the edible window period. Figure S3. Effects of Acetylsalicylic acid (ASA) treatment at different concentrations (0.5, 1.0 and 2.0 mmol/L) on the physiological quality of kiwifruit during the edible window period. Figure S4. Comparison of the expression patterns of selected DEGs obtained from RT-qPCR and RNA-Seq analysis.

## References

[1] Wang S., Qiu Y., Zhu F. (2021). Kiwifruit (*Actinidia* spp.): A review of chemical diversity and biological activities. Food Chem..

[2] Rakha A., Rehman N., Anwar R., Rasheed H., Rabail R., Bhat Z.F., Khaneghah A.M., Aadil R.M. (2025). *Actinidia* spp. (Kiwifruit): A Comprehensive Review of Its Nutraceutical Potential in Disease Mitigation and Health Enhancement. Food Front..

[3] Karagiannis E. (2024). Postharvest physiology of climacteric and nonclimacteric fruits and vegetables. Oxygen, Nitrogen and Sulfur Species in Post-Harvest Physiology of Horticultural Crops.

[4] Chai J., Wang Y., Liu Y., Yong K., Liu Z. (2021). 1-MCP extends the shelf life of ready-to-eat ‘Hayward’ and ‘Qihong’ kiwifruit stored at room temperature. Sci. Hortic..

[5] Yan H., Wang R., Ji N., Cao S., Ma C., Li J., Wang G., Huang Y., Lei J., Ba L. (2022). Preparation, shelf, and eating quality of ready-to-eat “Guichang” kiwifruit: Regulation by ethylene and 1-MCP. Front. Chem..

[6] Xia Y., Wu D., Ali M., Liu Y., Zhuang Q., Wadood S.A., Liao Q., Liu H., Gan R. (2024). Innovative postharvest strategies for maintaining the quality of kiwifruit during storage: An updated review. Food Front..

[7] Novais C., Molina A.K., Abreu R.M.V., Santo-Buelga C., Ferreira I.C.F.R., Pereira C., Barros L. (2022). Natural food colorants and preservatives: A review, a demand, and a challenge. J. Agric. Food Chem..

[8] Cedillo-Olivos A.E., Juárez-Chairez M.F., Cid-Gallegos M.S., Sánchez-Chino X., Jiménez-Martínez C. (2025). Natural preservatives used in foods: A review. Crit. Rev. Food Sci. Nutr..

[9] Blankenship S.M., Dole J.M. (2003). 1-Methylcyclopropene: A review. Postharvest Biol. Technol..

[10] Guo S., Xue K., Si S., Gu J., Liu L., Yan W., Zhang A., Ren P., Du X. (2025). Mechanisms of 1-Methylcyclopropene (1-MCP) in regulating ethylene signal transduction and its role in stress responses: A Review. Plant Sci..

[11] Huang H., Guo L., Wang L., Wang H., Ma S., Jiang Y., Qu H. (2019). 1-Methylcyclopropene (1-MCP) slows ripening of kiwifruit and affects energy status, membrane fatty acid contents and cell membrane integrity. Postharvest Biol. Technol..

[12] Xu F., Liu S., Liu Y., Xu J., Liu T., Dong S. (2019). Effectiveness of lysozyme coatings and 1-MCP treatments on storage and preservation of kiwifruit. Food Chem..

[13] Ma H., Liu N., Xie G., Tan S., Xu Y., Luo Z. (2025). Postharvest ripening combined with 1-MCP keeps the shelf quality of kiwifruit after cold storage by regulating the antioxidant system and defense enzymes. Postharvest Biol. Technol..

[14] Liu H., Pei H., Jiao J., Jin M., Li H., Zhu Q., Ma Y., Rao J. (2021). 1-Methylcyclopropene treatment followed with ethylene treatment alleviates postharvest chilling injury of ‘Xuxiang’ kiwifruit during low-temperature storage. Food Control..

[15] Huan C., Zhang J., Jia Y., Li S., Jiang T., Shen S., Zheng X. (2020). Effect of 1-methylcyclopropene treatment on quality, volatile production and ethanol metabolism in kiwifruit during storage at room temperature. Sci. Hortic..

[16] Chai J., Li J., Li L., Liao B., Liu Z. (2023). 1-MCP application in ‘Xuxiang’ kiwifruit at appropriate firmness avoids hard-core and prolongs the shelf life. Postharvest Biol. Technol..

[17] Giménez M.J., Serrano M., Valverde J.M., Martínez-Romero D., Castillo S., Valero D., Guillén F. (2017). Preharvest salicylic acid and acetylsalicylic acid treatments preserve quality and enhance antioxidant systems during postharvest storage of sweet cherry cultivars. J. Sci. Food Agric..

[18] Ze Y., Gao H., Li T., Yang B., Jiang Y. (2021). Insights into the roles of melatonin in maintaining quality and extending shelf life of postharvest fruits. Trends Food Sci. Technol..

[19] Madebo M.P., Zheng Y., Jin P. (2022). Melatonin-mediated postharvest quality and antioxidant properties of fresh fruits: A comprehensive meta-analysis. Compr. Rev. Food Sci. Food Saf..

[20] Xie Q., Zhang Y., Cheng Y., Tian Y., Luo J., Hu Z., Chen G. (2022). The role of melatonin in tomato stress response, growth and development. Plant Cell Rep..

[21] Kaygısız T., Sen F., Gundogdu M., Ejaz S., Aglar E. (2025). Effect of postharvest melatonin and salicylic acid applications on quality characteristics and primary and secondary metabolite physiology of tomato fruits. BMC Plant Biol..

[22] Hu W., Yang H., Tie W., Yan Y., Ding Z., Liu Y., Wu C., Wang J., Reiter R.J., Tan D.-X. (2017). Natural variation in banana varieties highlights the role of melatonin in postharvest ripening and quality. J. Agric. Food Chem..

[23] Liu S., Huang H., Huber D.J., Pan Y., Shi X., Zhang Z. (2020). Delay of ripening and softening in ‘Guifei’ mango fruit by postharvest application of melatonin. Postharvest Biol. Technol..

[24] Xue H., Sun Y., Li L., Bi Y., Hussain R., Zhang R., Long H., Nan M., Pu L. (2020). Acetylsalicylic acid (ASA) induced fusarium rot resistance and suppressed neosolaniol production by elevation of ROS metabolism in muskmelon fruit. Sci. Hortic..

[25] Arnao M.B., Hernández-Ruiz J. (2015). Functions of melatonin in plants: A review. J. Pineal Res..

[26] Gao H., Zhang Z.K., Chai H.K., Cheng N., Yang Y., Wang D.N., Yang T., Cao W. (2016). Melatonin treatment delays postharvest senescence and regulates reactive oxygen species metabolism in peach fruit. Postharvest Biol. Technol..

[27] Qu G., Wu W., Ba L., Ma C., Ji N., Cao S. (2022). Melatonin enhances the postharvest disease resistance of blueberries fruit by modulating the jasmonic acid signaling pathway and phenylpropanoid metabolites. Front. Chem..

[28] Zhang Y., Tang H., Lei D., Zhao B., Zhou X., Yao W., Fan J., Lin Y., Chen Q., Wang Y. (2023). Exogenous melatonin maintains postharvest quality in kiwiberry fruit by regulating sugar metabolism during cold storage. LWT.

[29] Luo Z., Zhang J., Xiang M., Zeng J., Chen J., Chen M. (2022). Exogenous melatonin treatment affects ascorbic acid metabolism in postharvest ‘Jinyan’ kiwifruit. Front. Nutr..

[30] Jiao J., Jin M., Liu H., Suo J., Yin X., Zhu Q., Rao J. (2022). Application of melatonin in kiwifruit (*Actinidia chinensis*) alleviated chilling injury during cold storage. Sci. Hortic..

[31] Yin X.-R., Zhang Y., Zhang B., Yang S.-L., Shi Y.-N., Ferguson I.B., Chen K.-S. (2013). Effects of acetylsalicylic acid on kiwifruit ethylene biosynthesis and signaling components. Postharvest Biol. Technol..

[32] Wang J., Zhu J.-Z., Liu X.-F., Allan A.C., Gao H.-Y., Yin X.-R., Wang W.-Q. (2022). Transcriptome analysis reveals mechanisms of acetylsalicylic acid-mediated fruit quality maintenance in fresh-cut kiwifruit. Postharvest Biol. Technol..

[33] Wang J., Liu X.-F., Zhang H.-Q., Allan A.C., Wang W.-Q., Yin X.-R. (2022). Transcriptional and post-transcriptional regulation of ethylene biosynthesis by exogenous acetylsalicylic acid in kiwifruit. Hortic. Res..

[34] Choi D., Choi J.H., Park K.-J., Kim C., Lim J.-H., Kim D.-H. (2023). Transcriptomic analysis of effects of 1-methylcyclopropene (1-MCP) and ethylene treatment on kiwifruit (*Actinidia chinensis*) ripening. Front. Plant Sci..

[35] Gundogdu M., Güler E., Ağlar E., Arslan T., Kan T., Çelik K. (2023). Use of spermidine to preserve organic acids, polyphenols, and quality of cold stored plum fruits. J. Food Compos. Anal..

[36] Tavarini S., Degl’iNnocenti E., Remorini D., Massai R., Guidi L. (2008). Antioxidant capacity, ascorbic acid, total phenols and carotenoids changes during harvest and after storage of Hayward kiwifruit. Food Chem..

[37] Zhang Y., Wang K., Xiao X., Cao S., Chen W., Yang Z., Shi L. (2021). Effect of 1-MCP on the regulation processes involved in ascorbate metabolism in kiwifruit. Postharvest Biol. Technol..

[38] Zhang X., Wang R., Zhang W., Chen C., Ma C., Ji N., Zhang N., Lei J., Tian Y., Zhang P. (2024). Microwave Treatment for Dictyophora rubrovolvata in Regulating Postharvest Autolysis and Energy Metabolism. Food Bioprocess Technol..

[39] Iqbal N., Khan N.A., Ferrante A., Trivellini A., Francini A., Khan M.I.R. (2017). Ethylene role in plant growth, development and senescence: Interaction with other phytohormones. Front. Plant Sci..

[40] Li L., Kitazawa H., Wang X., Sun H. (2017). Regulation of respiratory pathway and electron transport chain in relation to senescence of postharvest white mushroom (*Agaricus bisporus*) under high O2/CO2 controlled atmospheres. J. Agric. Food Chem..

[41] Cheng J., Zheng A., Li H., Huan C., Jiang T., Shen S., Zheng X. (2022). Effects of melatonin treatment on ethanol fermenation and ERF expression in kiwifruit cv. Bruno during postharvest. Sci. Hortic..

[42] Guo W., Zhang C., Yang R., Zhao S., Han X., Wang Z., Li S., Gao H. (2023). Endogenous salicylic acid mediates melatonin-induced chilling-and oxidative-stress tolerance in harvested kiwifruit. Postharvest Biol. Technol..

[43] Li M., Zheng Q., Lin H., Lin M., Chen Y., Lin Y., Fan Z., Wang H. (2020). The influence of ATP treatment on energy dissipation system in postharvest longan fruit during senescence. Postharvest Biol. Technol..

[44] Wang H., Qian Z., Ma S., Zhou Y., Patrick J.W., Duan X., Jiang Y., Qu H. (2013). Energy status of ripening and postharvest senescent fruit of litchi (*Litchi chinensis* Sonn.). BMC Plant Biol..

[45] Wang F., Yang Q., Zhao Q., Zhang X. (2020). Roles of antioxidant capacity and energy metabolism in the maturity-dependent chilling tolerance of postharvest kiwifruit. Postharvest Biol. Technol..

[46] Hu T., Zheng S., Liu Q., Li M., Chen J., Zhang H., Lin M., Lin H., Chen Y. (2025). Melatonin treatment maintains the quality properties and storability of carambola fruit by modulating energy metabolism. Food Chem..

[47] Morth J.P., Pedersen B.P., Buch-Pedersen M.J., Andersen J.P., Vilsen B., Palmgren M.G., Nissen P. (2011). A structural overview of the plasma membrane Na+,K+-ATPase and H+-ATPase ion pumps. Nat. Rev. Mol. Cell Biol..

[48] Azevedo I.G., Oliveira J.G., da Silva M.G., Pereira T., Corrêa S.F., Vargas H., Façanha A.R. (2008). P-type H+-ATPases activity, membrane integrity, and apoplastic pH during papaya fruit ripening. Postharvest Biol. Technol..

[49] Yi C., Qu H.X., Jiang Y.M., Shi J., Duan X.W., Joyce D.C., Li Y.B. (2008). ATP-induced changes in energy status and membrane integrity of harvested litchi fruit and its relation to pathogen resistance. J. Phytopathol..

[50] Yang Z., Cao S., Su X., Jiang Y. (2014). Respiratory activity and mitochondrial membrane associated with fruit senescence in postharvest peaches in response to UV-C treatment. Food Chem..

[51] Musatov A., Robinson N.C. (2012). Susceptibility of mitochondrial electron-transport complexes to oxidative damage. Focus on cytochrome c oxidase. Free Radic. Res..

