# Supplementary material for: Melatonin Extends the Edible Window Period of Kiwifruit by Modulating Energy Metabolism and Respiration: A Comparison with 1-Methylcyclopropene and Acetylsalicylic Acid
Source: Foods. 2026 Sep 29;15(19):3489. doi: 10.3390/foods15193489 (PMC13665305; doi:10.3390/foods15193489)
Supplement: Supplementary file 1 [file foods-15-03489-s001.zip › foods-4514165-supplementary.pdf]

Table S1. Primers used for RT-qPCR analysis of gene expression in this study

Gene	Forward Primer (5′-3′)
Actin	F: GCTTACAGAGGCACCACTCAACC
	R: CCGGAATCCAGCACCAATACCAG
PFK-6	F: TCCACTTCCATTCCGACCAA
(Actinidia32111)	R: TGGGTTAGGGTACGTAGGGA
iPGAM	F: CTGATGGCCGTGATGTCTTG
(Actinidia11471)	R:GCATCCCATCCACGTTTCAC
PDH E1β	F: GAGTTGTTGTATGGTGAGTC
(Actinidia30609)	R: CCTTTGCAAGGATCTCAGCT
CCO	F: GTATCCAAAGTTGCCACGGG
(Actinidia25935)	R:GTGGTTGGAAATCTCCAATC
PMA	F: AGAGATGGCTGGGATGGATG
(Actinidia08949)	R: CACGCTCGGCAAACTTATCA
ACLY	F: ACCTTGGCTCTCATCGGAAA
(Actinidia15066)	R: GCATCCTCCCATCTCAACCT

Table S2. Overview of transcriptome sequencing quality for 15 kiwifruit libraries across different treatment groups during the edible window period.

**Sample**	**Clean read number**	**Clean read radio (%)**	**Q30 (%)**
0d_1	45865610	98.48	95.72
0d_2	50014650	98.33	95.60
0d_3	54043696	98.52	95.66
CK11d_1	42776774	98.21	95.41
CK11d_2	46994612	98.30	95.32
CK11d_3	38562010	97.63	93.96
ASA11d_1	49393546	98.28	95.53
ASA11d_2	43795942	98.31	95.37
ASA11d_3	44336078	98.28	95.39
MT11d_1	50093100	98.37	95.45
MT11d_2	43758638	97.77	94.34
MT11d_3	40129144	98.44	95.47
1-MCP11d_1	44006318	98.42	95.64
1-MCP11d_2	41459468	98.33	95.61
1-MCP11d_3	50962486	98.30	95.65

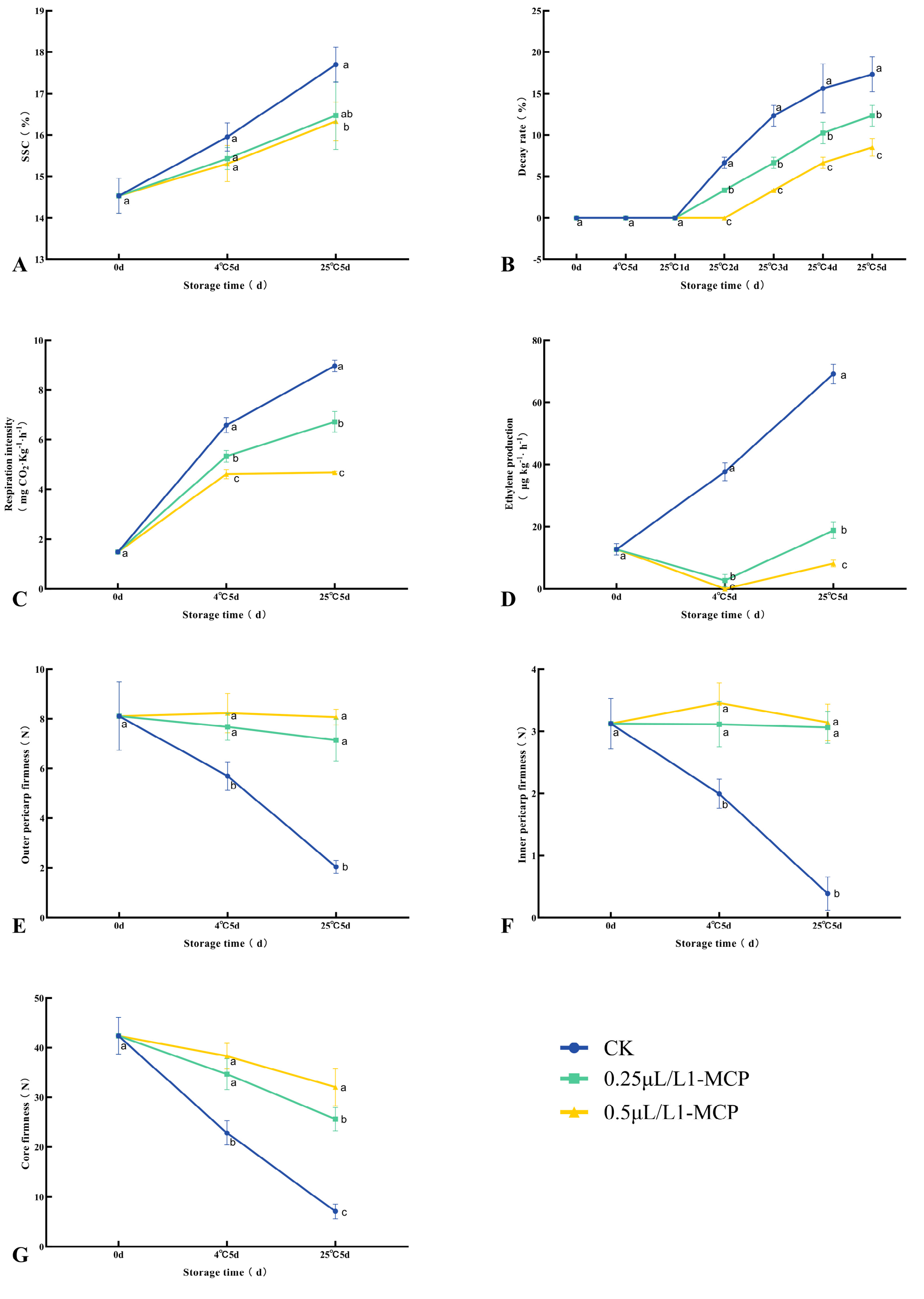

Figure S1. Effects of 1-methylcyclopropene (1-MCP) treatment at different concentrations (0.25 and 0.5 μL/L) on the physiological quality of kiwifruit during the edible window period. (A) SSC; (B) Respiration rate; (C) Ethylene production rate; (D) Decay rate; (E) Outer pericarp firmness; (F) Inner pericarp firmness; (G) Core firmness. Data represent the mean ± standard deviation ($n = 3$). Different lowercase letters indicate significant differences between treatments according to Duncan's multiple range test ($P < 0.05$).

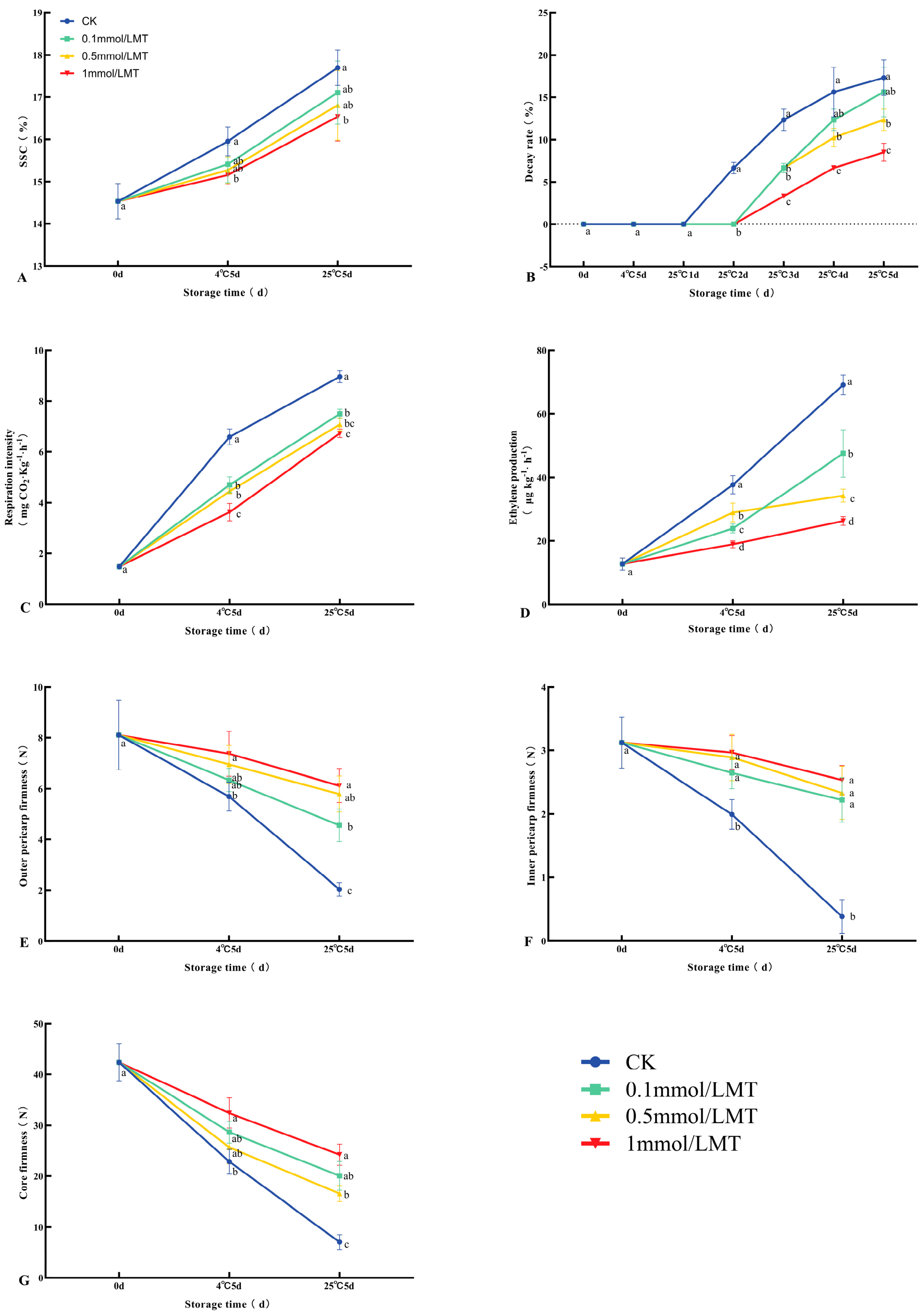

Figure S2. Effects of melatonin (MT) treatment at different concentrations (0.1, 0.5, and 1.0 mmol/L) on the physiological quality of kiwifruit during the edible window period. (A) SSC; (B) Respiration rate; (C) Ethylene production rate; (D) Decay rate; (E) Outer pericarp firmness; (F) Inner pericarp firmness; (G) Core firmness. Data represent the mean ± standard deviation (n = 3). Different lowercase letters indicate significant differences between treatments according to Duncan's multiple range test ($P < 0.05$).

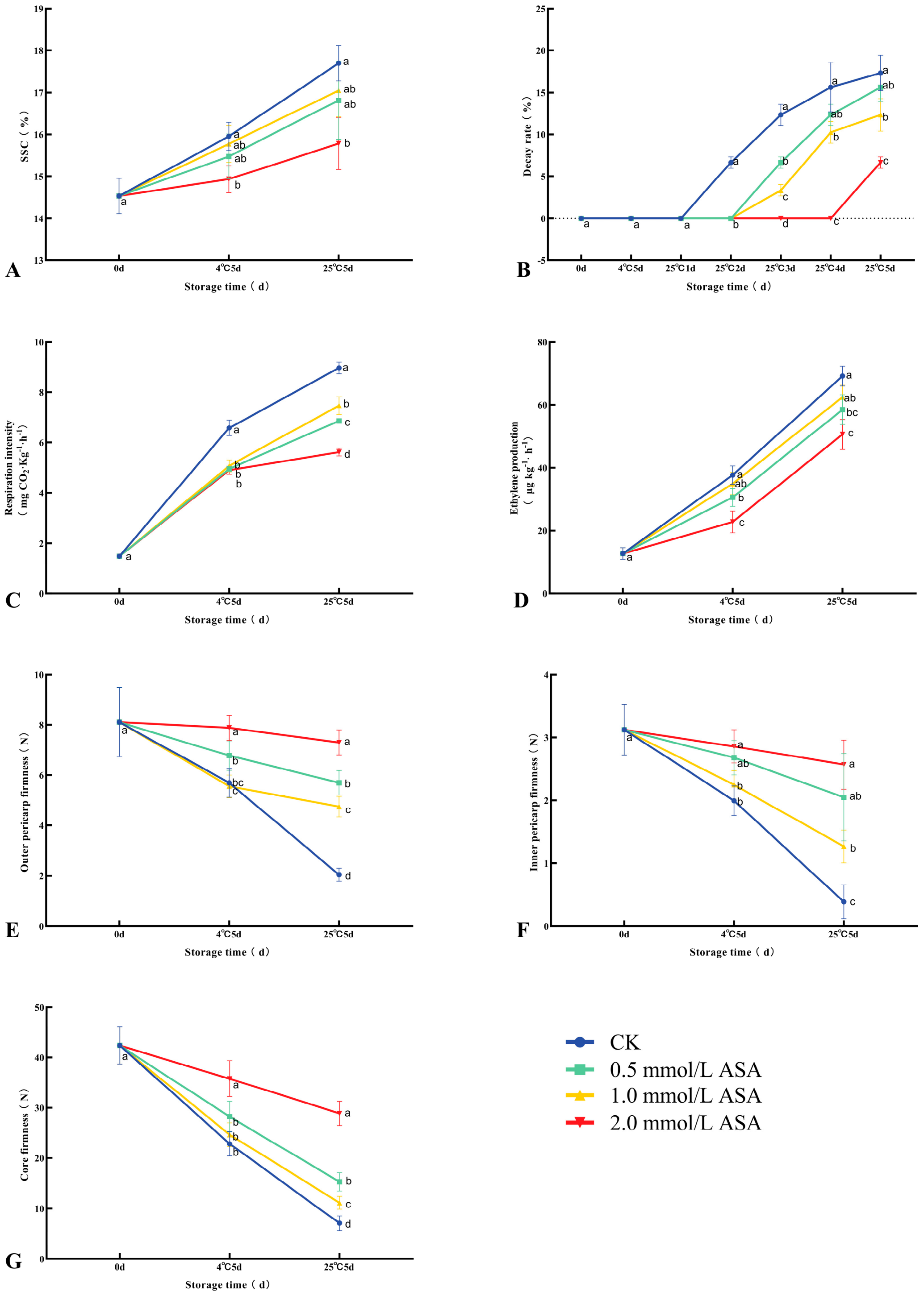

Figure S3. Effects of Acetylsalicylic acid (ASA) treatment at different concentrations (0.5, 1.0 and 2.0 mmol/L) on the physiological quality of kiwifruit during the edible window period. (A) SSC; (B) Respiration rate; (C) Ethylene production rate; (D) Decay rate; (E) Outer pericarp firmness; (F) Inner pericarp firmness; (G) Core firmness. Data represent the mean ± standard deviation ($n = 3$). Different lowercase letters indicate significant differences between treatments according to Duncan's multiple range test ($P < 0.05$).

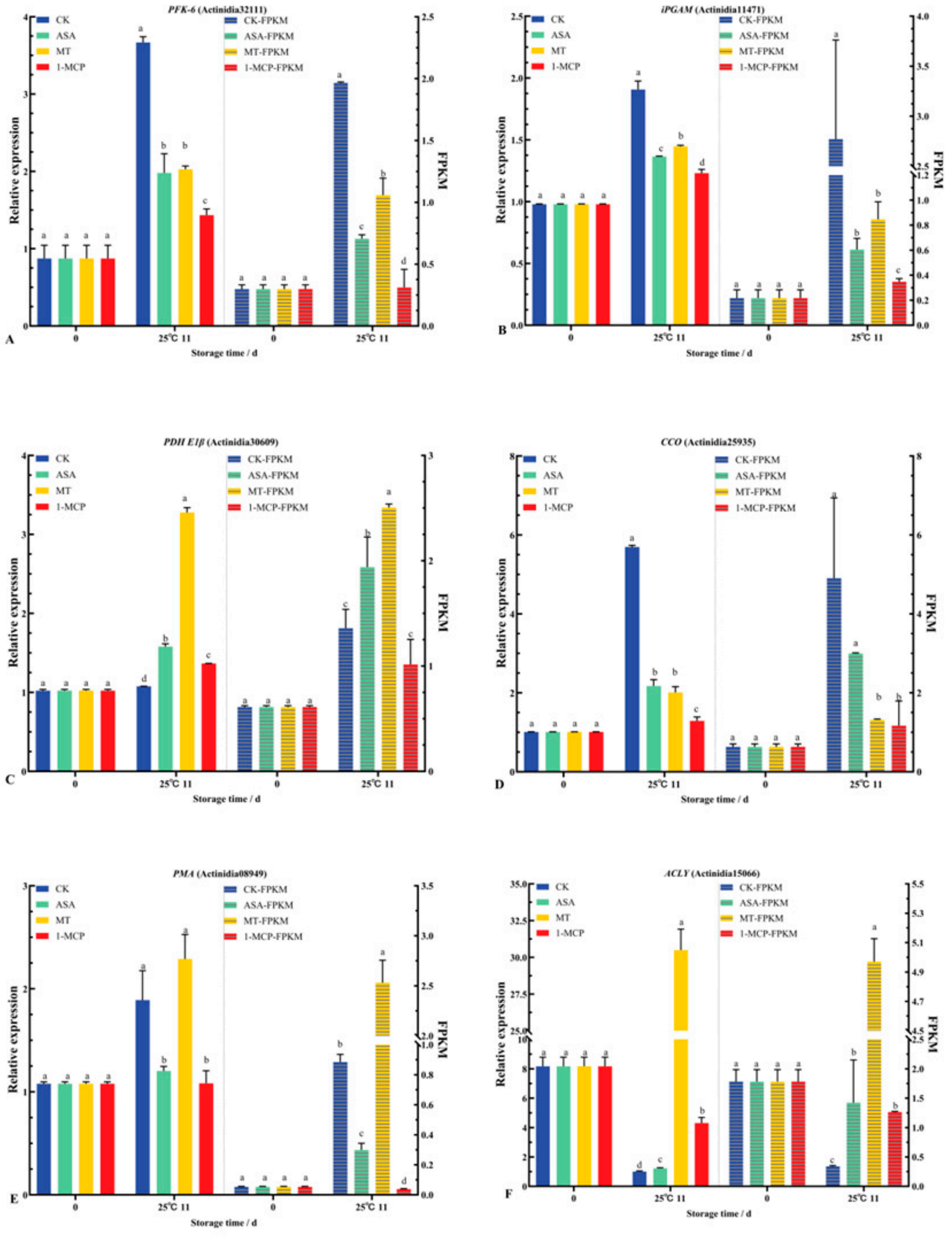

Figure S4. Comparison of the expression patterns of selected DEGs obtained from RT-qPCR and RNA-Seq analysis. The bars (left y-axis) represent the relative expression levels determined by RT-qPCR, and the hashed bars (right y-axis) represent the FPKM values from RNA-Seq data. Data represent mean ± standard deviation (n = 3). Different lowercase letters indicate significant differences between treatments per Duncan's test ($P < 0.05$).
